# Review on Microreactors for Photo-Electrocatalysis Artificial Photosynthesis Regeneration of Coenzymes

**DOI:** 10.3390/mi15060789

**Published:** 2024-06-15

**Authors:** Haixia Liu, Rui Sun, Yujing Yang, Chuanhao Zhang, Gaozhen Zhao, Kaihuan Zhang, Lijuan Liang, Xiaowen Huang

**Affiliations:** 1 Department of Bioengineering, Qilu University of Technology (Shandong Academy of Sciences), Jinan 250300, China; 10431211092@stu.qlu.edu.cn (H.L.); 10431211246@stu.qlu.edu.cn (Y.Y.); zhangchuanhao66@126.com (C.Z.); zhaogaozhen2021@126.com (G.Z.); 2 Jiaxing Key Laboratory of Biosemiconductors, Xiangfu Laboratory, Jiashan 314102, China; rsunsinap@163.com; 3 2020 X-Lab, Shanghai Institute of Microsystem and Information Technology, Chinese Academy of Sciences, Shanghai 200050, China; kzhang@mail.sim.ac.cn; 4 State Key Laboratory of Transducer Technology, Shanghai Institute of Microsystem and Information Technology, Chinese Academy of Sciences, Shanghai 200050, China; 5 Center of Materials Science and Optoelectronics Engineering, University of Chinese Academy of Sciences, Beijing 100049, China

**Keywords:** microreactor, artificial photosynthesis, regeneration of coenzymes, photo-electrocatalysis

## Abstract

In recent years, with the outbreak of the global energy crisis, renewable solar energy has become a focal point of research. However, the utilization efficiency of natural photosynthesis (NPS) is only about 1%. Inspired by NPS, artificial photosynthesis (APS) was developed and utilized in applications such as the regeneration of coenzymes. APS for coenzyme regeneration can overcome the problem of high energy consumption in comparison to electrocatalytic methods. Microreactors represent a promising technology. Compared with the conventional system, it has the advantages of a large specific surface area, the fast diffusion of small molecules, and high efficiency. Introducing microreactors can lead to more efficient, economical, and environmentally friendly coenzyme regeneration in artificial photosynthesis. This review begins with a brief introduction of APS and microreactors, and then summarizes research on traditional electrocatalytic coenzyme regeneration, as well as photocatalytic and photo-electrocatalysis coenzyme regeneration by APS, all based on microreactors, and compares them with the corresponding conventional system. Finally, it looks forward to the promising prospects of this technology.

## 1. Introduction

In recent years, with the aggravation of the energy crisis and environmental issues [1,2], there has been a significant interest in renewable energy collected from natural resources to replace fossil fuels [3,4,5,6]. Solar energy is ubiquitous, providing the earth with energy of approximately 10^17^ W [7,8], which is the most prevalent natural resource [9,10]. Since modern times, the use of solar energy has gradually increased, including applications such as photovoltaic power generation [11,12] and solar thermal collection [13,14]. Among them, photosynthesis is the most common method of solar energy collection and utilization [7]. Natural photosynthesis (NPS) is a process where green plants and some microorganisms capture and absorb light energy under suitable conditions [15,16]. Building upon the knowledge of NPS, a more efficient and flexible artificial photosynthesis system (APS) [17] has been developed. One of the research focuses is on obtaining reduced coenzyme I (NADH) by artificial photosynthesis to promote the production and preparation of fine chemicals. Redoxidoreductase is the general name of all enzymes that catalyze the redox reaction between two molecules. It is widely distributed in nature and participates in catalyzing various types of reactions to prepare a variety of chiral pharmaceutical intermediates, which promote the progress and development of the pharmaceutical industry [18,19,20]. At the same time, oxidoreductase also participates in the key steps of biological cell metabolism and energy conversion [21,22], and has considerable research potential in the preparation of sor and environmental treatment fields [23,24,25]. However, in the application of oxidoreductase, a coenzyme such as NADH is often needed as a hydride donor to promote the reaction towards catalysis [26]. However, the high price of coenzymes limits the large-scale application of oxidoreductase. Therefore, the construction of an efficient and cheap coenzyme regeneration system is a key link to realize the industrialization of the enzymatic process of high-value fine chemical synthesis [27]. Current methods for coenzyme regeneration predominantly include enzymatic, chemical, photocatalytic, electrocatalytic, and photoelectric catalysis methods based on conventional systems.

Microfluidics is a technology that integrates the steps of sample preparation, mixing, separation, purification, and detection in chemical reactions into a micro platform in a flexibly controllably manner. Compared with conventional systems, microreactors have the advantages of a high specific surface area, high mass and heat transfer efficiency, low sample consumption, and high reaction efficiency. Therefore, with the help of a microreactor, it is expected to realize green, efficient, cost-effective, flexible, and controllable coenzyme regeneration, and at the same time, this will pave a new way for research into artificial photosynthesis. This paper primarily summarizes the research work on photocatalytic, electrocatalytic, and photo-electrocatalytic coenzyme regeneration based on both conventional systems and microfluidic systems in recent years. It provides an overview of artificial photosynthesis and microfluidic technology, and it discusses the opportunities and challenges that the future development of microfluidic coenzyme regeneration will face, serving as a reference for professionals in this field.

## 2. Artificial Photosynthesis and Coenzyme Regeneration

Artificial photosynthesis is a new energy technology developed based on natural photosynthesis, and it has great potential to replace fossil fuels to alleviate the global energy crisis by the sustainable production of solar fuel through APS [28]. At present, coenzyme regeneration is one of the important applications of APS. The following will briefly introduce artificial photosynthesis and coenzyme regeneration.

### 2.1. Principle of Artificial Photosynthesis

As a research hotspot at present, renewable energy includes solar energy, geothermal energy, water energy, ocean temperature difference energy, air kinetic energy, ocean surface wave energy, biomass storage energy, periodic tidal energy, and so on [29,30]. They are recycled and regenerated on the earth, a rich reserve energy that can be used by human beings continuously to play the roles of power supply, heating, and transportation [5], and can be replenished and regenerated automatically without deliberate participation. As the most important part of renewable energy, solar energy is an effective way to relieve the problems of the energy crisis [31], food shortage [32], and climate change [33].

Photosynthesis is one of the core chemical reactions in the world [34,35], which is the basis of energy conversion and material circulation in nature and maintains the stability and balance of the ecosystem [36,37,38]. NPS fixes carbon dioxide (CO_2_) and releases oxygen through photosynthesis to realize the conversion from solar energy to chemical energy, which is an extremely important way to capture and utilize solar energy. The realization of natural photosynthesis is mainly divided into two steps: light reaction and dark reaction.

Artificial photosynthesis based on NPS can decompose water by absorbing light energy to produce clean hydrogen, and absorb CO_2_ to achieve the purpose of CO_2_ fixation. The construction of photocatalytic APS is mainly divided into the simulation of PSII and PSI of photosynthetic systems [39]. Compared with NPS, APS can utilize a wider spectrum of solar energy, and can be divided into three types according to specific energy sources: photochemical (PC), photo-electrochemistry (PEC), and photovoltaic electrochemistry (PV-EC) [40]. However, whether it is NPS or APS, the acquisition and utilization of clean and sustainable solar energy includes three basic functions, namely, light capture, charge separation, and redox catalysis [41]. 

**Figure 1:**
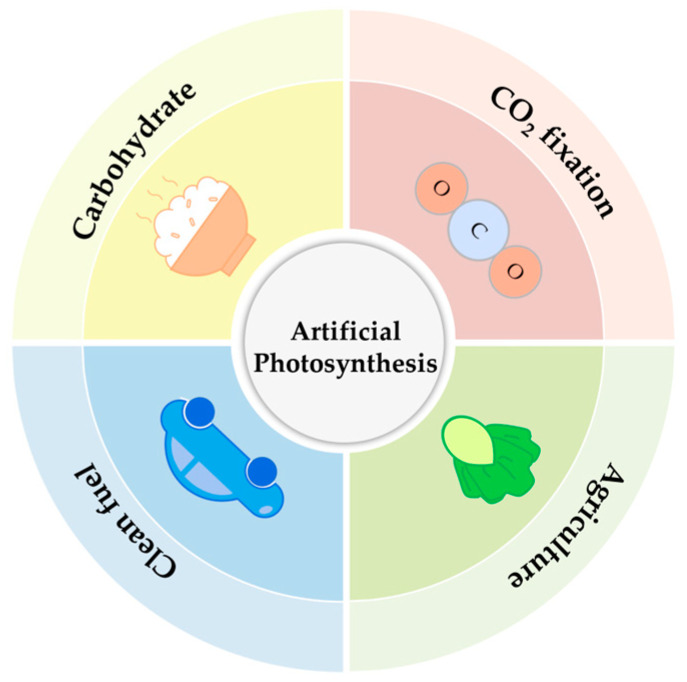
Application field of artificial photosynthesis.

On the one hand, artificial photosynthesis cannot be limited by the reaction environment; on the other hand, it can break through the low efficiency of natural photosynthesis [42]. At the beginning of the 19th century, people carried out related research on the conversion and synthesis of chemical energy based on artificial photosynthesis [43] and achieved important research work, such as the photolysis of water [44]. Up to now, artificial photosynthesis mainly has the following development and application directions (Figure 1): the production of clean fuel [45], CO_2_ fixation [46,47,48], the conversion of carbohydrates [49], and the promotion of agricultural production [50,51].

### 2.2. Coenzyme Regeneration

Catalytic reaction is an efficient way to synthesize high-value chemicals from raw materials [52]. However, in many cases, additional by-products such as stereoisomers will be produced in the process of catalytic reaction with traditional catalysts, which makes it necessary to separate and purify the products after the reaction, which not only prolongs the reaction period but also increases the production cost. It was found that, different from traditional catalytic reactions, biocatalytic reactions using enzymes have a high specificity, which can selectively synthesize stereospecific isomers needed by the reactions, reduce the generation of additional by-products, and realize the large-scale production of chemicals [53,54], among which oxidoreductase, which accounts for about 25% of the discovered enzymes in the world, naturally becomes the research focus. Redoxidoreductase is the general name of all enzymes that catalyze the redox reaction between two molecules. It is widely distributed in nature and participates in catalyzing various types of reactions to prepare a variety of chiral pharmaceutical intermediates, which promote the progress and development of the pharmaceutical industry [18,19,20]. At the same time, oxidoreductase also participates in the key steps of biological cell metabolism and energy conversion [21,22], and plays an important role in the preparation of biosensor and environmental treatment, with considerable research potential [23,24,25]. In the application of oxidoreductase, a coenzyme such as NADH is often needed as a hydride donor [26]. NADH is an important product produced by the citric acid cycle during the glycolysis and respiration of biological cells, in which N stands for nicotinamide, A stands for adenine, and D stands for dinucleotide. NADH plays an important role in the process of material circulation and energy metabolism in cells, and it also participates in energy transfer to promote ATP synthesis, which plays an important role in cell growth, differentiation, and metabolism. In the biocatalytic reaction of oxidoreductase, the coenzyme NADH plays the role of reducing equivalents and pushes the reaction towards catalysis [55]. However, the high price and high consumption of coenzymes limit the large-scale application of oxidoreductase [56]. Therefore, the construction of an efficient, cheap, and environmentally friendly coenzyme regeneration system is a key link to realize the industrialization of the enzymatic process to synthesize high-value chemicals on a large scale [27].

**Figure 2:**
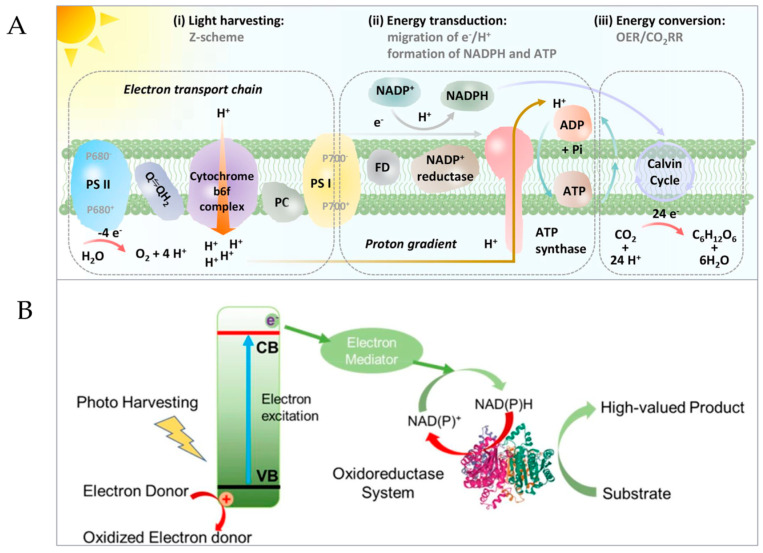
Schematic diagram of coenzyme regeneration. (**A**) Natural photosynthesis systems [40]. (© 2023 Wiley-VCH GmbH). (**B**) Artificial photosynthesis systems [39]. (Copyright © 2021, Jiangnan University).

The important factor of coenzyme regeneration is the hydrogen anion (H+2e^−^), and the key step is to transfer one proton and two electrons to β-Nicotinamide adenine dinucleotide (β-NAD^+^) for reduction, so as to obtain the target product coenzyme NADH. In NPS, under the irradiation of sunlight, chlorophyll in Photosynthetic System II (PSII) absorbs light energy to generate excitation electrons. PSII and Photosynthetic System I (PSI) work together in Z-mode, and photogenerated electrons are transferred to PSI through the Z-mode electron transfer chain, reducing NAD(P)^+^ to NAD(P)H, thus storing solar energy in chemical bonds (Figure 2A) [57].

In the photocatalysis and photo-electrocatalysis of coenzyme regeneration based on artificial photosynthesis, the photoreaction stage of NPS corresponds to the PSII simulation of APS, and the photosensitizer is used to crack electron donors to produce oxygen and hydrogen as clean energy. The dark reaction stage of NPS is a PSI simulation corresponding to APS, and the light reaction stage is also simulated. Specifically, the photocatalytic material in APS is illuminated, and the photocatalytic material absorbs light energy to generate photo-excited electrons, some of which jump to the surface of the photocatalyst and are then transported through electron transfer mediators to realize coenzyme regeneration, and the regenerated coenzyme can be subsequently coupled with enzymes to catalyze and generate high value-added chemicals (Figure 2B). Especially in the photo-electric catalytic coenzyme regeneration, the external circuit can further promote the reaction to the direction of target coenzyme NADH production and improve the efficiency of coenzyme regeneration.

## 3. Microreactors

Microfluidic technology was born from the research of microanalysis and first appeared in capillary form [58]. Using microfluidic technology, biochemistry, materials science, mechanical dynamics, and other disciplines can combine to realize the integrated reaction of sample pretreatment, separation, and detection [59], and it has been applied in many fields such as drug screening and synthesis [60], cell research [61], life science [62] and micro-particle synthesis [63].

### 3.1. Preparation Materials of Microreactor

The common preparation materials of microreactors are as follows: silicon, polydimethylsiloxane (PDMS), glass, high-temperature-resistant ceramics, polymethylmethacrylate (PMMA), metal, paper base, cloth base, etc., in addition to wood base, polypropylene (PP), hydrogel, polystyrene (PS), and so on. Their selection is determined by the reaction conditions [64].

1. Silicon

**Figure 3:**
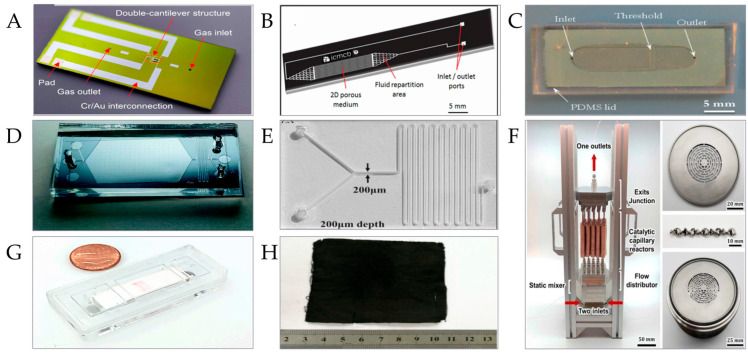
Materials of microreactor. (**A**) Silicon-based microreactor [65]. (Copyright © 2022, American Chemical Society) (**B**) Glass-based microreactor [68]. (Copyright © 2020, Science and Technology of Advanced Materials) (**C**) Ceramic-based microreactor [70]. (Copyright © 2009 Elsevier B.V. All rights reserved). (**D**) PDMS-based microreactor [73]. (Copyright © 2022, Royal Society of Chemistry) (**E**) PMMA-based microreactor [75]. (Copyright © 2020, Nano Express). (**F**) Metal-based microreactor [79]. (Copyright © 2019, Royal Society of Chemistry). (**G**) Paper-based microreactor [84]. (Copyright © 2020, Royal Society of Chemistry). (**H**) Cloth-based microreactor [89]. (Copyright © 2018, Royal Society of Chemistry).

Silicon material is the first material used to prepare microreactors, and its preparation process is mature, with a high manufacturing accuracy, good repeatability, stable properties, and high-temperature and high-pressure resistance, which is suitable for the preparation of various microreactors. Yao et al. developed a silicon-based microreactor for the simultaneous analysis of TGA-TEM (Figure 3A), which can synchronously and accurately analyze the thermal stability and microscopic characterization of materials, and has obvious advantages in precision, accuracy, and detail [65]. In addition, silicon has semiconductor characteristics and infrared transparency. As reported by Susarrey-Arce and others, they used silicon as the main material of the microreactor, and then carefully discussed the influence of applying an electric field on the gas or liquid medium in the flow channel of the microreactor, demonstrating their ability to track the liquid phase reaction in situ [66].

2. Glass

Glass is low in cost and easy to obtain, and also has the advantages of high hardness, high-temperature resistance, good chemical stability, and good biocompatibility. The high light transmittance of glass is also suitable for preparing optical microreactors. Liu et al. reported that they successfully prepared a 2D B_2_O_3_ crystal/CsPbBr_3_ composite with excellent optical properties and good stability by making a glass microreactor with borophosphate glass, which is expected to play an important role in the photo-electric field [67]. As well as visible light, glass microreactors can also be used for the in situ characterization of mid-infrared. Ari et al. developed a novel germanate glass with wide light transmittance and then prepared a microreactor by anodic bonding technology (Figure 3B), which was used to realize the in situ characterization of visible light and mid-infrared [68].

3. Ceramic

Ceramics have the advantages of high strength, ultra-high temperature resistance, excellent chemical stability, high hardness, corrosion resistance, and so on. They can withstand extremely high reaction temperatures and are suitable for a variety of high-temperature chemical reactions. Liao et al. prepared a porous SiC ceramic-based microreactor with the advantages of oxidation resistance, corrosion resistance, and high-temperature resistance by a solution combustion method. With this microreactor, methanol steam reforming with an efficiency as high as 100% can be realized for the preparation and extraction of clean hydrogen [69]. Some ceramics can also be used in micro-electronics instead of silicon-based materials. Malecha et al. manufactured an enzyme microreactor with good stability by using low-temperature co-fired ceramics (LTCC) technology (Figure 3C), which integrated a heater and a temperature sensor and obtained urea detection results similar to those of silicon-based microreactors at low cost [70].

4. PDMS

PDMS is a high molecular polymer and one of the most commonly used materials for microreactor preparation. It has the advantages of easy processing, low cost, heat and cold resistance, aging resistance, stable viscosity, high permeability, high elasticity, electrical insulation, good thermal conductivity, non-toxicity, odorlessness, good biocompatibility, and good chemical stability. Based on the above advantages, a PDMS microreactor can be used to prepare a variety of products. Adiyala et al. realized the synthesis of high-value fused imidazole derivatives in high yield by preparing a PDMS microreactor with a ruthenium immobilized catalyst [71]. A PDMS microreactor can also be used for portable detection on site. Zengerle et al. reported a real-time PDMS microreactor for a reverse transcription polymerase chain reaction experiment, and portable A-H3N2 virus detection can be fully realized automatically by using this PDMS microfluidic chip [72]. A PDMS microreactor can also be used in infrared spectroscopy to monitor the reaction process in real-time. Lozeman et al. prepared a high-resolution PDMS microreactor (Figure 3D) with soft lithography technology, which is simple and easy to repeat. Based on this microreactor, they carried out the Paal–Knorr reaction at a low cost, showing its great potential in infrared detection applications [73]. However, in general, strong alkali often cause corrosion loss to PDMS. In a strong alkaline environment, polydimethylsiloxane may be broken by siloxane bonds under the action of an acid-base catalyst, which will damage PDMS. This property limits the experimental research and industrial application of PDMS in strong alkaline conditions, and it still needs people to explore it [74].

5. PMMA

Large aspect ratio channels with a low processing loss can be prepared by PMMA. PMMA has the advantages of easy processing, a low price and affordability, high permeability, corrosion resistance, good chemical stability, and the like, and ultraviolet visible light can pass through it, so that it is widely used in the fields of building materials, medical equipment, aerospace, optical instruments, and the like. At the same time, PMMA also has the bright spots of weatherability and processability. Prakash et al. reported that they prepared a PMMA microreactor with a laser engraving machine (Figure 3E). ZnS quantum dots with a high dispersibility, uniform size, and excellent photocatalytic activity were successfully synthesized in this microreactor, and the excellent antibacterial activity of the prepared ZnS quantum dots was verified by Escherichia coli test [75]. Different from PDMS, PMMA has the advantage of being easy to keep clean and is more suitable for preparing a self-priming compartmentalization (SPC) microreactor. Jin et al. prepared a new SPC microreactor without a complex microfluidic structure and with a low risk of cross-contamination, which can quickly realize multiple nucleic acid amplification to accurately detect a variety of DNA [76].

6. Metal

Metal materials have good mechanical properties, high-temperature and high-pressure resistance, and are suitable for high exothermic reactions. Li et al. designed and prepared a stainless steel and platinum segmented high energy density microreactor with a combustion chamber, and optimized the micro-reaction quickly and accurately by the Kriging method. This microreactor has considerable potential to replace lithium-ion batteries as the energy source for portable electronic products [77]. Metal microreactors can also be used for high-performance catalytic reactions. Kiwi-Minsker et al. reported a structured catalytic wall microreactor (SCWMR) composed of two aluminum plates with a structured catalyst sandwiched between them. Different from the higher pressure drop caused by gas passing through the catalyst in the common sandwich reactor, the reaction gas in SCWMR is transferred to the catalytic layer by diffusion, so the microreactor can achieve a two to three orders of magnitude pressure drop reduction and carry out selective high-performance acetylene hydrogenation [78]. In addition, the metal microreactor can also be used for Qualcomm quantity drug synthesis. Ahn et al. prepared a stainless steel microreactor (Figure 3F) with 3D printing and numerical control processing technology. The microreactor body was used as the catalyst, which avoided the problems of a high pressure drop and catalyst leaching in other catalyst fixation methods, and the microreactor had a good mixing effect and successfully synthesized anticonvulsants with a high yield [79].

7. Paper

Paper-based materials have the advantages of being cheap and easily available, with good flexibility, strong self-transport ability, etc., and they are mostly used with trace chemical substances and biological analysis [80], the early diagnosis of diseases, and other research work. Thuy et al. reported a new paper-based substrate with a simple operation, environmental protection, and no surfactant. They used an inkjet microreactor to deposit silver nanoparticles on office paper pre-modified by chitosan, to obtain a paper-based substrate with surface-enhanced Raman scattering (SERS). The paper-based substrate showed excellent sensitivity and point-to-point reproducibility in the field detection and analysis application scene of portable Raman spectrometers, and its repeatability and stability made it very suitable for real-time testing applications [81]. There are also studies on biocatalytic synthesis based on paper-based microreactors [82]. In addition, paper-based microreactors can also be used for electrochemical detection and microchip platform electrophoresis [83]. Hasan et al. developed a new HemeChip hemoglobin detection system, which is based on the electrophoresis process of a paper-based microreactor (Figure 3G). It is low in cost and easy for large-scale production. It is suitable for many standard blood sample collection methods such as fingertip blood collection, and the detection results can be obtained in 10 min without professional operation, which is beneficial for the rapid early screening and real-time tracking detection of hemoglobin diseases, reducing the risk of hemoglobin diseases and helping to protect human health around the world [84].

8. Cloth

A cloth-based microreactor uses easily available cloth substrate as a preparation material and uses capillary force between cloth-based fibers to drive the test solution, which is mostly used for sensing analyses such as colorimetry and chemiluminescence. A cloth base has the same advantages as a paper base, such as strong conveying ability, easy processing, good biocompatibility, easy degradation, and easy carrying, which greatly reduces the reaction difficulty and cost, and it has considerable application potential in medical diagnosis, biochemical research, and flexible product research and development [85,86]. Wang et al. obtained a modified fabric microreactor by firmly growing zinc oxide (ZnO) nanostructures on the surface and inside of the modified cotton fiber by a chemical deposition method. The microreactor has the advantages of excellent heat resistance and ultraviolet blocking performance, and because the ZnO grown on it is not simply coated on the outer surface of the microreactor but firmly deposited in the fiber, it is resistant to friction and cleaning and can be applied to the practical development of outdoor clothes and shelter products, reducing the threat to human health caused by excessive ultraviolet radiation [87]. In addition, ZnO can also be used to study the enzyme-mediated regeneration of NDA+. Ottone et al. demonstrated that polycrystalline ZnO nanostructures are effective materials for the photo-electrochemical regeneration of NAD^+^. Applying a voltage to the electrode induces NADH near the electrode lose electrons and transforms it into NAD+. Furthermore, increasing illumination further improves the efficiency of NADH oxidation and the regeneration of NAD^+^, and ultimately demonstrates the excellent enzyme activity of NAD^+^ regenerated by FDH reaction [88]. And cloth-based microreactors can also be used in environmental remediation research. Luo et al. reported an economical and simple technology to prepare a new material (Co_3_O_4_-incorporated carbon composite) by modifying fiber sheets with cobalt complexes. The continuous cloth-based microreactor (Figure 3H) designed based on this material has the ability to remove bisphenol A(BPA) and is expected to be applied to the treatment of industrial organic wastewater [89].

### 3.2. Preparation Technology of Microreactor

At present, the preparation technology of microreactors mainly includes standard lithography technology, soft lithography technology, 3D printing technology, textile technology, laser-induced hydrophilization technology, screen printing technology, thermoforming technology, spacing technology, machining, and so on.

1. Standard Lithography Technology

**Figure 4:**
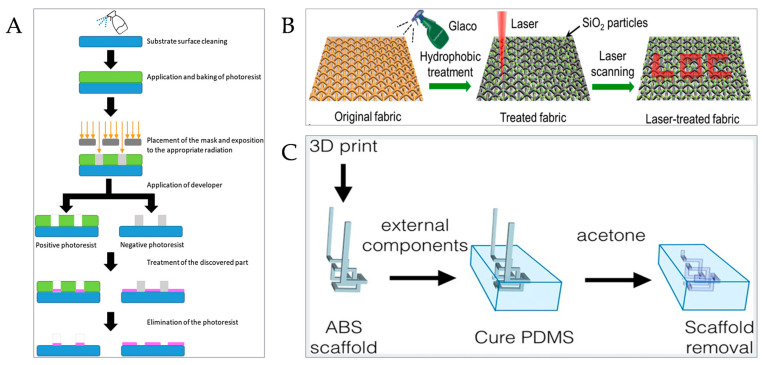
Preparation technology of microreactor. (**A**) Photolithography preparation steps [92]. (© 2021 The Authors. Published by Elsevier B.V. on behalf of Institution of Chemical Engineers). (**B**) Laser-induced hydrophilization technology [97]. (Copyright © 2021 Elsevier B.V. All rights reserved). (**C**) Preparation step of partition method [102]. (© 2015 The Authors. Published by WILEY-VCH Verlag GmbH & Co. KGaA, Weinheim).

Standard lithography technology is the most common microreactor preparation technology in the current research work, which is suitable for the preparation of microfluidic chips with various microfluidic channels and structural types [90]. The specific steps (Figure 4A) include substrate cleaning, photoresist spin coating, soft baking, photoresist exposure, developer development, film hardening, etching, and residue removal [91,92].

2. Soft Lithography Technology

Soft lithography is a common method to make PDMS microreactors, and its main steps include mask design and fabrication, the spin coating of silicon wafer photoresist, photoresist exposure, the immersion development of developer, etching, residue removal, pouring, chip packaging, and so on. Compared with standard lithography technology, the microreactor prepared by soft lithography technology has a higher precision, and the preparation process can meet the flexible requirements of the reaction. This technology can be used for the preparation of multi-layer and three-dimensional microfluidic reactions [93]. Konda et al. reported that they used soft lithography technology to prepare a double-layer elastic retractable microreactor with reversible sealing advantages. Using this reactor and a solution phase method, conductive copper traces of target patterns can be deposited on planar and non-planar substrates simply, cheaply, and flexibly [94].

3. 3D Printing

Complex microreactors can be prepared by using 3D printing technology, which is flexible in operation, directly molded, and environmentally friendly. Consumables for 3D printing mainly include polymers, metals, ceramics, glass, and so on. Alimi et al. reported that they prepared a new microreactor containing an immobilized palladium nano-catalyst by 3D printing technology, and used it to study the heterogeneous catalysis of morin dye in a continuous flow system [95].

4. Textile Technology

The application of textile technology is in the preparation of a cloth-based microreactor. Its preparation steps mainly include silk yarn pretreatment and manual/textile machine preparation, in which the predetermined flow path of its fluid is realized by the difference between the hydrophilicity and hydrophobicity of silk yarn. Bhandari et al. reported that silk yarns and jacquard accessories with different hydrophilicity were selected to construct the target fluid flow path in the fabric in the textile machine, and proposed to adjust the absorption capacity of the fabric by using parameters such as yarn twist and weaving area, and finally obtain a fabric containing loaded reagents for the concept verification immunoassay and carry out actual detection through visual reading [96].

5. Laser-Induced Hydrophilization Technology

In this technology, a hydrophobic barrier is formed by coating a hydrophobic solution (e.g., silica nanoparticle solution) on the fabric, and then a super-hydrophilic target micro-pattern is constructed on the fabric with the super-hydrophobic surface by using a focused laser, to obtain the fabric microreactor. Its principle is to preset the laser scanning to change the contact angle at a specific position of the cloth base. Xu et al. used laser-induced hydrophilization technology to prepare a cloth-based microfluidic analysis device, which can be used for rapid, convenient, and low-cost real-time blood type testing and analysis (Figure 4B) [97].

6. Screen Printing Technology

Screen printing technology has the advantages of convenience, quickness, easy operation, and the small space occupied by instruments. The principle is that hydrophobic materials such as wax and polymer are selectively loaded on the cloth base by preparing the printing plate with the required microfluidic structure pattern, and the corresponding hydrophobic barrier pattern is formed on the surface of the cloth base to obtain the expected microreactor. Tasaengtong et al. reported that they used cheap and common polystyrene as a hydrophobic material and realized the one-step preparation of a cloth-based microreactor by simple, fast, and low-price screen printing technology [98]. This technology has the advantages of high reproducibility on various substrates, simple operation, convenience, quickness, flexibility, and controllability, and does not need any expensive equipment such as ovens and hot plates, which reduces the preparation cost of microreactors and at the same time avoids additional tedious steps such as heating and shortens the preparation time.

7. Thermoforming Technology

This technology takes advantage of the deformation characteristics of glass and other materials by heating and adopts pressing, blowing, and other methods to obtain a microreactor with a microstructure. Its manufacturing process is simple, the preparation requirements are low, and the time consumption is short. Taking glass material as an example, after heating and softening the glass, the viscosity of the glass increases rapidly with the decrease in temperature, and the glass can be molded and self-adhered, cooled, and hardened, so that a solid microreactor can be obtained, and it can also be vacuum-sealed by non-evaporable getter [99]. Eklund et al. used thermoforming technology to prepare microsphere cavities. They etched deep holes in the silicon wafer, then bonded them to form a sealed cavity, and then the glass material, softened at high temperature, was prepared into a spherical cavity shape through gas expansion in the sealed cavity. This technology can be used to prepare miniature glass housings with high-precision requirements, which can also be used to prepare microlenses on a large scale [100].

8. Spacer Technique

Spacer technology can be used to prepare microreactors with special internal structures. The main steps are preparing erasable spacers and embedding them in the base material, and after the base material is completely cured, removing the pre-embedded spacers to obtain the target microreactor [101]. Using this technology, there is no need for a sealing operation, which improves the preparation efficiency [92]. There is no need for additional lithography equipment, which simplifies the reaction steps and reduces the reaction cost [102]. Saggiomo et al. used acrylonitrile butadiene styrene as spacer material and acetone as a spacer elimination reagent to prepare three-dimensional and multi-layer complex microchannels on a single PDMS substrate by a simple two-step scaffold elimination technique (Figure 4C) [102].

9. Mechanical Processing

Machining technology is a commonly used method of microreactor preparation, which is mostly used for the microreactor preparation of glass and other substrate materials, with a simple operation, low equipment requirements, and mature technology. This technology usually uses special tools and abrasives to cut and polish the microstructure on the reactor substrate to obtain the microreactor. Plaza et al. cut and prepared a prismatic target pattern microstructure with a high aspect ratio and high verticality on a glass substrate by simple mechanical sawing, and then bonded the glass anode with the prismatic structure to a silicon substrate to obtain a glass–silicon microreactor with good sealing performance and excellent heat insulation [103].

The preparation method of the microreactor is flexibly selected according to the different materials, application environment, and precision requirements (Table 1). Choosing the appropriate preparation method helps to maximize the advantages of the microreactor and obtain the best observation effect and experimental results in the reaction process.

### 3.3. Application Fields of Microreactors

At present, microreactors are mostly used in many biomedical fields, such as enzyme immobilization, cell research, tumor screening, particle synthesis, and nano-morphology research [104].

1. Immobilization of Enzymes

A microreactor is a powerful tool for the study of enzyme immobilization [105]. The immobilization of an enzyme can be realized by combining the enzyme with the carrier in a microreactor by physical and chemical methods, which can prevent the enzyme from dissociating in the reaction system and improve the maintenance of enzyme activity. The immobilization of enzymes in a microreactor improves its biological activity and stability, simplifies the recovery of products and enzymes, improves its repeatability and stability, and realizes a continuous reaction, which can greatly shorten the time consumption of detection and analysis, reduce the consumption of experimental materials, and reduce the reaction cost. It promotes the research of drug screening [106].

2. Cell Research

**Figure 5:**
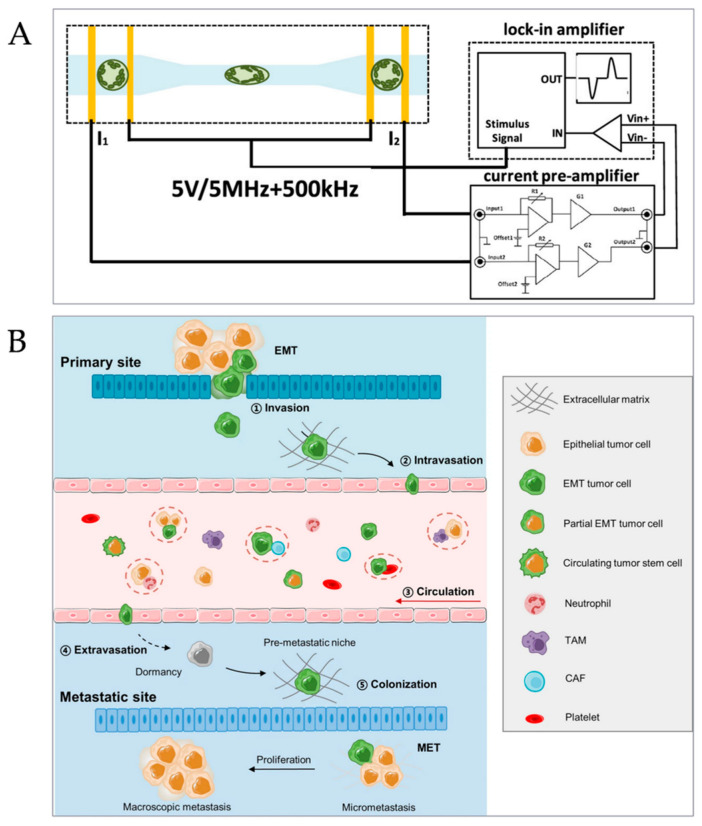
Application fields of microreactors. (**A**) Microfluidic flow cytometer [107]. (Copyright © 2020, Anal. Chem). (**B**). Cancer metastasis process [108]. (Copyright © 2021, Signal Transduct. Target. Ther).

Using the high integration of a microreactor and the flexibility of channel design, it is convenient to realize a variety of biochemical analyses. On the one hand, because the size of microchannels is mostly close to the size of biological cells, the precise investigation and control of biological single cells can be realized. On the other hand, the microfluidic chip can simulate the internal environment of the human body to study three-dimensional cell characteristics. Furthermore, by controlling the flow bed of the reagent in the microreactor, the sorting of cells can be realized, and the labeling treatment of cells is omitted. Hand et al. reported a microfluidic resistance flow cytometer for studying the special biological and physiological properties of individual plant cells (Figure 5A), which can be used to characterize the mechanical and electrical characteristics in a single plant cell at the same time, and also to verify the role of auxin [107].

3. Tumor Screening

Cancer metastasis is a complex process including the steps of cancer cells invading the primary site, joining the circulation, circulating to survive and interact with blood cells, extravasation from the circulation, metastasis and attachment, etc. The specific steps are shown in the following figure (Figure 5B) [108]. Among them, circulating tumor cells (CTCs) are the main cause of death from biological malignant tumors. Using a microreactor to screen CTCs in vivo is beneficial to early tumor screening and late dynamic real-time detection, and it improves the survival rate of patients. Different from the traditional CTC sorting method, the high-purity, high-Qualcomm content, high-enrichment, and real-time-monitoring sorting of CTC cells can be realized by using the high efficiency, visibility, and flexible controllability of a microreactor, which greatly facilitates the biomedical research work of tumor cell screening [109].

4. Synthesis of Tiny Particles

The microreactor can accurately control the micro-upgraded volume of fluid containing tiny particles in a highly integrated reaction environment. The efficiency of fluid mixing and separation is improved by adding plentiful types of main forces such as inflatable pressure, magnetism, and dielectric power into the microreactor [110], which is beneficial to the preparation and formation of tiny particles such as fluorescent microspheres and microcarrier droplets, to promote the further development of drug delivery and catalyst loading [111,112]. Compared with conventional biochemical reaction systems, a microreactor reaction system has the following advantages: (1) High efficiency. Thanks to the advantages of microreactors, such as micro-reaction volume, a high specific surface area, and high mass and heat transfer efficiency, the reaction time is greatly shortened, and the reaction efficiency is improved. (2) Low cost and small reagent consumption. (3) Using the flexibility and operability of the microreactor, the synthesized fine particles have a high flux and good uniformity and quality. (4) A good sealing performance, not easily influenced by the external environment, reducing the evaporation of the reaction system in the reaction process, avoiding the influence of non-human factors on the reaction results, and realizing accurate control of the reaction system.

5. Food Safety Inspection

Food safety detection using a microreactor has the advantages of high sensitivity, high specificity, high stability, high accuracy, easy carrying, low time consumption, and low cost. Microreactors have achieved excellent application results in food safety detection for *Salmonella*, *Clostridioids*, mycotoxins, and pesticide residues [113,114,115]. Chen et al. prepared a microreactor by immobilizing acetylcholinesterase on the inner wall of the capillary with a coating layer of dopamine, which can be used to stably determine organophosphorus pesticide residues in fruits and vegetables [116]. The microreactor can also be connected with mobile phones and apps to analyze the test results more conveniently and quickly. Zhang et al. built an OP pesticide detection platform by embedding a microreactor into a microfluidic paper-based analysis device. The platform has the advantages of high sensitivity and high repeatability on the basis of good stability and can be applied to the actual detection of OP residues in food [117].

6. Drug Screening

The research and development cost and time cost of new drugs are extremely high. Among them, the rapid screening of drug efficacy and toxicity before the clinical trial stage can reduce the cost and shorten the time [118,119]. Furthermore, microreactors can readily simulate the three-dimensional environment of cells in the human body, and the gap between the research and development evaluation of new drugs before and after the clinic can be reduced by using them for drug screening [120]. Moreover, microreactors can also be used to screen the absorption effect of drugs. At present, the main methods of drug administration are oral administration, intramuscular injection, and intravenous injection [121], among which oral administration is the most commonly used. Using a microreactor can simulate the release and absorption process of drugs in the stomach and intestines, and evaluate and optimize the absorption effect of new drugs.

## 4. Coenzyme Regeneration in Conventional System and Microreactor System

Redoxidoreductase is an important enzyme used in the chemical industry. Coenzymes such as NADH are often needed to promote a reaction towards the synthesis of target catalytic products. However, the high price and high consumption of coenzymes limit the industrial application of oxidoreductase. Therefore, the construction of an efficient, low-cost, and environment-friendly coenzyme regeneration system is of great research significance for realizing the industrialization of the enzymatic synthesis of high-value chemicals. The addition of a microreactor is beneficial to improve the efficiency of the coenzyme regeneration reaction, avoid the step of catalyst recovery, reduce the generation of by-products, improve the quality of target coenzyme products, and finally realize a more economical, friendly, and environmentally friendly coenzyme regeneration.

At present, the research work on coenzyme regeneration is mainly based on the enzymatic method, chemical method, photocatalytic method, electrocatalytic method, and photo-electric catalytic method. The difference between the study of coenzyme regeneration based on microreactors and the conventional system is shown in Table 2. The recent research work on the latter three in the conventional system and microreactor system will be summarized.

### 4.1. Photocatalytic Coenzyme Regeneration

As a current research hotspot, photocatalytic coenzyme regeneration stands out because of its clean and cheap advantages. Its function usually requires the existence of a photocatalyst and electron/proton transport medium [122]. As an important utilization of artificial photosynthesis, the regeneration of coenzymes by the APS system can reduce the cost and price of coenzymes, promote the industrial application of oxidoreductase, and promote the large-scale production of fine chemicals and medical drugs.

The steps of photocatalytic coenzyme regeneration are mainly divided into three parts: Firstly, the dispersed/fixed photocatalyst in the photocatalytic system is irradiated to absorb light energy and generate photogenerated electrons. Then, some of these electrons will be excited to the conduction band (CB), leaving corresponding holes in the valence band, and some of them will recombine with the holes. Some photogenerated electrons will jump to the surface of the catalyst, and two photogenerated electrons and a single proton will be transferred to NAD^+^ through the electron transfer medium. Finally, NAD^+^ obtains electrons and protons to realize the regeneration of the coenzyme NADH. Excess photogenerated holes are consumed by sacrificial agents such as water and triethanolamine (TEOA), which further improves the efficiency of photocatalytic coenzyme regeneration.

**Figure 6:**
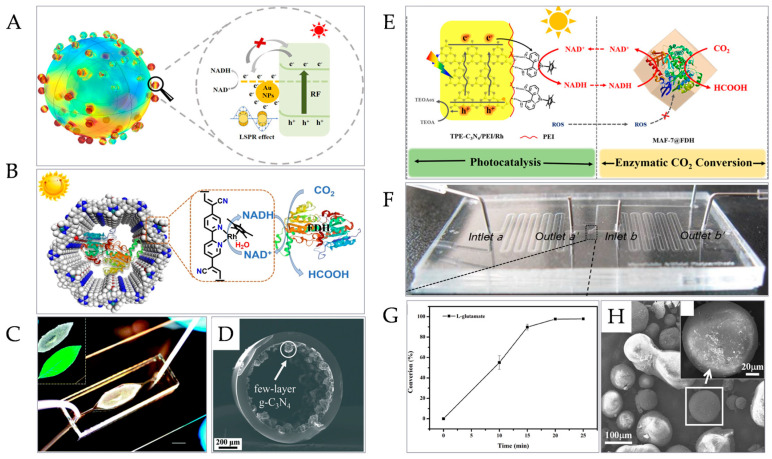
Photocatalytic coenzyme regeneration. (**A**) HRF-Au hollow nanosphere photocatalyst [125]. (Copyright © 2022, American Chemical Society). (**B**) Dual-functional novel core–shell nano-reactor [127]. (Copyright © 2022, Wiley-VCH GmbH) (**C**) Blade-like photocatalytic microreactor [129]. (Copyright © 2016, The Royal Society of Chemistry). (**D**) Capillary photocatalytic microreactor [132]. (Copyright © 2022, Catalysis Science & Technology). (**E**) Inorganic photocatalyst–enzyme system for formic acid synthesis [133]. (Copyright © 2020, American Chemical Society). (**F**) Photoreaction zone–dark reaction zone integrated microreactor [134]. (Copyright © 2011, The Royal Society of Chemistry). (**G**) Synthesis of L-glutamic acid [135]. (© 2022, Elsevier B.V. All rights reserved). (**H**) Photocatalytic microcapsule reactor [136]. (Copyright © 2018, Nanomaterials).

In the study of photocatalytic coenzyme regeneration, the research focus is mainly divided into two parts: First, to explore and synthesize new photocatalysts such as organic dyes, metal oxidants, covalent organic frameworks (COFs), and semiconductor photocatalysts [123,124], and use them as electron-driven carriers to improve coenzyme regeneration efficiency or optimize reaction conditions and reduce reaction costs. The other is to construct a new system based on photocatalysis, photo-electrocatalysis, and photo-enzyme coupling catalysis to realize coenzyme regeneration. The development of an efficient photocatalyst is an important research direction to improve the regeneration efficiency of photocatalytic coenzymes. Zhou et al. successfully prepared resorcinol-formaldehyde resin nanospheres modified by gold nanoparticles (HRF-Au) by the hard template method and photo-deposition (Figure 6A). Compared with pure resorcinol-formaldehyde resin nanospheres, the regeneration rate of NADH under visible light irradiation was improved by four times [125]. Wang et al. prepared two kinds of conjugated microporous polymer (CMP) photocatalysts connected by thiazole [5,4-d] thiazole (TZZ) units to carry out high-efficiency coenzyme regeneration, and integrated with three continuous enzymes to construct a CMP–enzyme mixing system, realizing the continuous conversion of CO_2_ into methanol with high selectivity [126]. Lin et al. reported a technology of coupling MIL-125-NH_2_ with an Rh complex, which promoted the realization of photocatalytic continuous coenzyme regeneration with an efficiency as high as 66.4%, and produced high-value formic acid by fixing formate dehydrogenase and coupling it with a coenzyme regeneration system [122]. At present, most of the regeneration of photocatalytic coenzymes begins with the delivery of photogenerated electrons to an Rh complex. Zhao et al. prepared a novel porous solid carrier by connecting a well-designed mesoporous olefin with the COF (Figure 6B) and used this carrier to co-immobilize formate dehydrogenase (FDH) and an Rh complex; photocatalytic coenzyme regeneration can be promoted by adjusting the addition amount of the latter, and the apparent quantum yield is about 9.17%, far exceeding a coenzyme regeneration photocatalyst composed of various crystal materials. Moreover, the constructed photocatalyst–enzyme coupling system has the ability to realize the selective, efficient, and continuous conversion of CO_2_ to high-value products [127]. Wu et al. reported that they successfully prepared a conjugated microporous polymer DTS/Rh@CMPs, which has excellent photostability and biocompatibility and can be used to realize the efficient photocatalytic regeneration of the coenzyme NADH, and studies have proved that intramolecular electron transfer is more effective than intermolecular electron transfer in photocatalytic reaction [124]. Ma et al. prepared a new metal-free photocatalyst PDA/g-C_3_N_4_ by the characteristic that poly-dopamine (PDA) spontaneously oxidizes and polymerizes on the surface of graphite carbonitride (g-C_3_N_4_) in an alkaline environment to form a functional coating. It was found that the coating of PDA effectively promoted the separation and delivery of photo-generated electron–hole pairs in g-C_3_N_4_. Under visible light irradiation, an efficient coenzyme NADH regeneration can be realized by using PDA/g-C_3_N_4_, and its coenzyme yield is six times higher than pure g-C_3_N_4_ [128].

Photocatalytic coenzyme regeneration based on a microreactor has also been reported. Compared with the conventional system, photocatalytic coenzyme regeneration in a microreactor has the advantages of easy recovery of the catalyst and high photocatalytic efficiency. The preparation of the reactor usually requires the following steps: the immobilization of the photocatalyst and the synthesis and introduction of the electron transfer medium. Huang et al. reported that the prepared photocatalyst graphite-phase carbon nitride (g-C_3_N_4_) and electron transfer medium (M) were highly integrated into the same microfluidic chip in one step (Figure 6C), and up to 63% coenzyme regeneration was achieved under the irradiation of light. Compared with the conventional bulk g-C_3_N_4_ suspension system and few-layer g-C_3_N_4_ suspension system, its coenzyme regeneration reaction is 23 times and 2.3 times faster, simplifying the preparation steps and improving the reaction efficiency [129]. In addition, the semiconductor in the microreactor can be modified with cocatalysts such as nanoparticles and monatomic species to further improve the photocatalytic performance of the microreactor; for this purpose, metal catalysts are common. The combination of metal and semiconductor can inhibit the recombination of photogenerated electrons and holes, and can also change the light absorption range and enhance light absorption, leading to a better photocatalytic effect [130]. For instance, a modified Pt monatomic metal cocatalyst (Pt SA) can further improve the utilization rate of Pt to obtain a better photocatalytic effect, while maintaining the original advantages of Pt in reducing the recombination rate of electron–hole pairs of TiO_2_. Rej et al. reported that they had prepared an ultra-thin titanium dioxide bronze nanosheet (TiO_2_ -BNS). A large number of dispersed Pt SA can be formed by virtue of the 2D morphology and sufficient high-strength coordination sites of the nanosheets, and the photocatalytic efficiency of the nanosheets modified with Pt SA can be improved by 10 times and 99 times, respectively, under solar illumination and 365 nm UV-LED illumination [131]. The structural design of the microreactor is also the key point to improving efficiency and reducing reaction costs. Lin et al. used low-cost and easily available glass capillaries to construct a microreactor (Figure 6D). Using the viscosity and nonspecific adhesion of PDMS before curing, the photocatalyst was fixed in the glass capillary conveniently, cheaply, and firmly, and the regeneration of the coenzyme NADH was as high as 56.03% [132]. Photocatalytic microreactors can also be coupled with enzyme-catalyzed reactions to realize the preparation of sustainable high-value chemicals. Tian et al. established a new kind of inorganic photocatalyst–enzyme system with a functional compartment (Figure 6E). While realizing coenzyme regeneration, the FDH was encapsulated in a metal–organic framework (MOF) to avoid inactivation during light excitation, and the conversion from CO_2_ to high-value formic acid was successfully carried out [133]. Lee et al. designed and prepared a microreactor containing both a photoreaction area with the photocatalyst immobilized and a dark reaction area with GDH immobilized (Figure 6F), to realize coenzyme regeneration and coupling to realize the synthesis of L- glutamic acid [134]. Wang et al. designed a blade-like microreactor composed of flexible waterproof double-sided adhesive tape and glass with strong sealing, which has good stability and high repeatability, and realized the regeneration of the coenzyme NADH with an efficiency of 87.19% and the synthesis of L-glutamic acid with a conversion rate of 97.65% (Figure 6G) [135]. In addition, photocatalytic coenzyme regeneration based on a cellular functional microreactor was also studied. Lin et al. used amphiphilic modified TiO_2_ nanoparticles (NPs) as the assembly component of a microcapsule membrane by the Pickering emulsion method to prepare photocatalytic microcapsules (Figure 6H), encapsulated alcohol dehydrogenase (ADH), and then modified them with poly-dopamine (PDA) to obtain a bionic ADH@TiO_2_NP microreactor to realize microfluidic photocatalytic coenzyme regeneration, and the generated coenzyme NADH was provided to the ADH for enzymatic reaction [136].

In addition, besides the cell-like microreactor, there is also research work on photocatalytic coenzyme regeneration based on cells. Yang et al. realized the photocatalytic coenzyme regeneration of artificial cells by artificially constructing cells containing cyanobacteria that can collect solar energy, fix CO_2_, convert light energy into chemical energy, and then successfully oxidize the prepared NADH under the action of lactate dehydrogenase to obtain lactic acid products. This research work has solved the key problems of artificial cells for energy supply and metabolic simulation. It laid a solid foundation for the further study of energy supply and metabolism in artificial cells and promoted the understanding and research of a cell working mechanism and evolution [137].

### 4.2. Electrocatalytic Coenzyme Regeneration

Electrocatalytic coenzyme regeneration is an important method of coenzyme regeneration, which is easy to construct and inexpensive. Using electrocatalysis, the coenzyme can be regenerated, and the CO_2_ can be fixed and transformed quickly. With the expansion of human activities and influence, the global carbon cycle balance has been continuously destroyed. While using clean energy such as solar energy to replace traditional fossil fuels to alleviate this situation, using simple and renewable continuous electrocatalysis to fix carbon dioxide can also play an important role in maintaining the carbon cycle balance, and its products have an ideal application value, such as common formate (HCOOH), which has a high energy value, is easy to handle and transport, easy to store, and non-toxic. On the one hand, it can be used as liquid fuel or a fuel additive; on the other hand, it can also be used for power generation. Different from the chemical method, the electrocatalytic method does not need a complex reducing agent to participate in the reaction, and it is mainly divided into two types: direct electrocatalytic coenzyme regeneration and indirect electrocatalytic coenzyme regeneration. In the late 20th century, Elving et al. reported that a direct electrocatalytic coenzyme regeneration was realized on an electrode without additional materials and surface modification, and they expounded the principle of NAD^+^ electroreduction into the coenzyme NADH.

**Figure 7:**
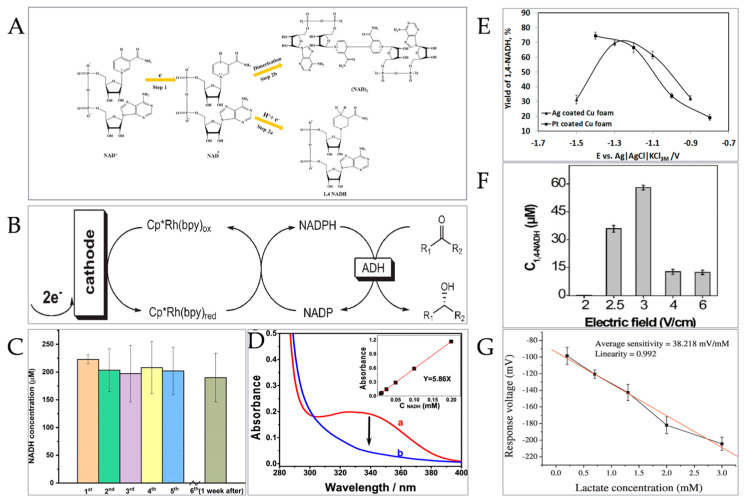
Electrocatalytic coenzyme regeneration. (**A**) Direct electrocatalytic reaction principle [55]. (Copyright © 2020 Wiley-VCH GmbH). (**B**) Indirect electrochemical cofactor regeneration [140]. (Copyright © 2008 WILEY-VCH Verlag GmbH & Co. KGaA, Weinheim). (**C**) Stability test results [141]. (Copyright © 2019 American Chemical Society). (**D**) UV–vis absorption spectra of products obtained before (a) and after (b) adding alcohol dehydrogenase and acetaldehyde [144]. (Copyright © 2022, ACS Appl. Mater. Interfaces). (**E**) A schematic diagram of the influence of electrocatalytic potential on the regeneration yield of coenzyme [146]. (© 2017 Elsevier Ltd. All rights reserved). (**F**) A schematic diagram of the influence of electric field on the regeneration yield of coenzyme [147]. (© 2021 Wiley-VCH GmbH). (**G**) Average sensitivity and linearity of array-flexible lactic acid enzymatic biosensor [148]. (© 2017 Elsevier Ltd. All rights reserved).

Direct electrocatalysis is simple in structure and convenient to operate, but it requires high potential to regenerate coenzymes, and it is easy to generate additional by-products such as NAD_2_ dimer and 1,2-NADH on the electrode surface (Figure 7A), and the required regeneration efficiency of the 1,4-NADH coenzyme is reduced [55,138,139]. It is found that electrode modification by depositing modified metal particles on the electrode surface can improve the efficiency and selectivity of coenzyme regeneration, but its repeatability is poor, and it is not suitable for continuous coenzyme regeneration. In indirect electrocatalytic coenzyme regeneration, electrons and protons are transported through electron transfer media such as the Rh complex, which reduces the generation of additional by-products and improves the coenzyme regeneration efficiency.

Hildebrand et al. developed two new mediators, Cp*Rh (5,5′- methyl −2,2′- bipyridine) and Cp*Rh (4,4′- methoxy −2,2′- bipyridine) (Figure 7B), and obtained results of electrocatalytic coenzyme regeneration with a volume productivity as high as 136 mmol L^−1^ d^−1^ and a turnover frequency as high as 113 h^−1^ [140]. Yuan et al. reported that they constructed a system for immobilizing myocardial yellow enzyme with Cc-PAA polymer, thereby achieving high-stability coenzyme regeneration with a 97–100% yield and 78–99% Faraday efficiency (Figure 7C) [141]. Alkotaini et al. prepared a modified glassy carbon electrode with the ability to regenerate the coenzyme NADH and used it to regenerate the coenzyme NADH at least eight times during the 16 h reaction by bio-electrocatalysis [142]. Zhang et al. prepared coordination compounds by the covalent binding of the bipyridine ligand to the electrode surface, and then reacting it with pentamethyl cyclopentadienyl rhodium (III) chloride dimer. The immobilized catalyst can be applied to the stable electrocatalytic regeneration of the coenzyme NADH, and its Faraday efficiency is as high as 87% [143]. Li et al. prepared NU-1000 thin film grown on glassy carbon to obtain a functional electrode with adjustable electrocatalytic activity, and used this electrode to realize the regeneration of the highly selective coenzyme NADH with a Faraday efficiency of 97% (Figure 7D) [144]. In addition, the electrode potential in electrocatalytic coenzyme regeneration will also affect the regeneration rate of the coenzyme, and the yield ratio of by-product /NADH during coenzyme regeneration is highly dependent on the change in potential [145]. Barin et al. prepared copper foam electrodes loaded with silver and platinum, respectively, by a galvanic cell replacement method and then studied the relationship between the electrocatalytic potential of the electrode and the regeneration yield of coenzyme under optimal deposition conditions (Figure 7E). The results showed that the required yield of 1,4-NADH first increased with the decrease in negative potential, and then continued to decrease with the decrease in potential after obtaining a yield of about 70% [146].

The research work of combining microfluidic technology with the electrocatalytic method to realize high-efficiency coenzyme regeneration is also one of the hotspots. The introduction of microfluidic technology greatly improves electrocatalytic efficiency and reduces the generation of coenzyme regeneration by-products. O‘Brien et al. controllably bound ADH on the surface of a microelectrode and used the hydroxide generated by a microelectrode discharge to influence the local pH value of the microenvironment and adjust it to the pH value suitable for enzyme activity, thus promoting the catalytic reaction of ADH to reduce local NAD^+^ to NADH and realize electrocatalytic coenzyme regeneration. The microelectrode can also be used to start other enzyme activities at predetermined positions [149]. The relationship between the electric field of the microelectrode and the results of coenzyme regeneration was also studied. Zhang et al. used an electrode fixed with a rhodium complex to carry out electrocatalytic coenzyme regeneration for 90 min. The result (Figure 7F) showed that the target coenzyme NADH was not synthesized at a low electric field value. When the electric field value exceeded 2.5 V/cm, the coenzyme regeneration product 1,4-NADH was produced, and the transformation amount increased with the increase in electric field intensity. When the electric field was increased to 4 V/cm, the product 1,4-NADH decreased [147]. Microfluidic electrocatalytic coenzyme regeneration can also be combined with enzymatic reaction for biological detection. Chou et al. used 3-glycidyl propyl trimethoxysilane (GPTS) to immobilize L-lactate dehydrogenase (LDH) and NAD^+^ on NiO thin films with a high chemical stability and high electrocatalysis, to realize coenzyme regeneration and further catalyze the enzymatic reaction of lactic acid, and successfully prepared an array-flexible lactic acid biosensor with an average sensitivity of 38.218 mV/mM (Figure 7G) [148].

### 4.3. Photo-Electrocatalysis Coenzyme Regeneration

Photocatalytic coenzyme regeneration is realized by the photo-electrocatalytic system (PEC) under the irradiation of light and external bias. A photo-electric catalytic coenzyme regeneration system usually consists of a photo-electrode, electrolyte, and external circuit. Different from the electrocatalytic coenzyme regeneration method, photo-electrocatalysis uses electrons excited by a photo-electrode to reduce NAD^+^, in which the photo-electrode for the oxidation reaction is called the photo-anode and the photo-electrode for the reduction reaction is called the photocathode. The main process is that under illumination, the photo-electrode absorbs the irradiated light energy to generate photo-induced electrons and holes, and at the same time, the bias voltage is added through an external circuit to promote the separation of photo-induced electron and hole pairs and the transmission of photo-induced electrons, and then the generated electrons are transferred to NAD^+^ by electron transmission media such as [Cp*Rh(bpy)(H)]^+^(M) to realize photo-electrocatalytic coenzyme regeneration.

**Figure 8:**
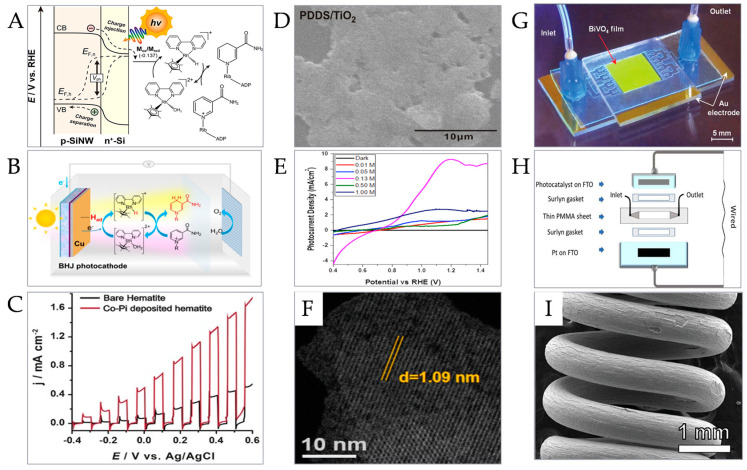
Photo-electrocatalysis coenzyme regeneration. (**A**) Regeneration scheme of coenzyme in photocathode [150]. (Copyright © 2023, J. Am. Chem. Soc.). (**B**) Schematic diagram of photocatalytic coenzyme regeneration in photocathode in presence of M [151]. (Copyright © 2024, American Chemical Society) (**C**) Linear sweep voltammetry (LSV) scanning characterization of hematite before and after Co-Pi deposition under visible light chopping illumination. [152]. (© 2017, Wiley-VCH Verlag GmbH & Co. KGaA, Weinheim) (**D**) SEM characterization of ITO conductive glass modified by PDDS/TiO_2_ film [153]. (Copyright © 2011, Elsevier B.V. All rights reserved.) (**E**) LSV scanning curve of PFP-PAs under condition of no light and illumination [154]. (© 2020, Hydrogen Energy Publications LLC. Published by Elsevier Ltd. All rights reserved.) (**F**) HAADF-STEM characterization of metal–organic frameworks [155]. (Copyright © 2022, American Chemical Society) (**G**) Physical diagram of photo-electric catalytic microreactor [156]. (Copyright © 2012, The Royal Society of Chemistry) (**H**) Schematic diagram of continuous photo-electrocatalytic microreactor [157]. (Copyright © 2019, The Royal Society of Chemistry) (**I**) SEM image of anodic titanium oxide coil [158] (© 2016, The Korean Society of Industrial and Engineering Chemistry. Published by Elsevier B.V. All rights reserved).

In the photo-electrocatalysis method, by applying external bias, a better coenzyme regeneration effect can be achieved, which is difficult to achieve by simple photocatalysis or electrocatalysis. In the 1970s, Fujishima and others realized the photo-electric decomposition of water through rutile single-crystal electrodes by using photo-electric catalysis technology [43]. However, there are still many problems with photo-electrocatalysis technology, such as low photocathode photovoltage, which leads to the need for a high cathode bias to realize coenzyme NADH regeneration. To solve this problem, Lineberry et al. reported a method of photo-electrocatalytic coenzyme regeneration by a photocathode (n^+^p-SiNW) with a high light voltage (Figure 8A). The prepared photocathode absorbs light energy to generate photogenerated carriers and a high light voltage of 435 mV. Because the quasi-Fermi energy level of electrons is negative to the redox potential of [Cp*Rh(bpy)H_2_O]^2+^(M_OX_) M_OX_/[Cp*Rh(bpy)H]^+^(M_red_), the photogenerated electrons are transferred to M_OX_, and finally, the M_red_-mediated conversion rate of 1.63 μmol H^−1^cm^−1^ is achieved at 0.2 V_RHE_ [150]. Liu et al. proposed a general strategy of coupling a proton reduction electrocatalyst and regioselective molecular catalyst (Figure 8B) and successfully realized an efficient (photo-) electrocatalytic regeneration of NADH by using a Cu-modified polymer matrix heterojunction (BHJ) photocathode and M complex [151]. Kuk et al. designed a series PEC battery with a hematite photocathode and bismuth ferrite photocathode based on iron oxide (Figure 8C). Under light irradiation, the battery used water as the electron donor to regenerate the coenzyme NADH [152]. There are also many research reports on the development of new electrodes. Ho et al. grew a poly (4,4′-diaminodiphenyl sulfone)/TiO_2_ (PDDS/TiO_2_) composite film on indium tin oxide (ITO) conductive glass (Figure 8D), and innovatively measured the photo-electrocatalysis ability of the PDDS/TiO_2_ composite film, which proved the excellent photo-electrocatalysis response of the composite film. The electrode modified by the PDDS/TiO_2_ composite film realized a high efficiency, high sensitivity, and high efficiency [153].

Compared with the conventional system, photo-electric catalytic coenzyme regeneration based on a microreactor has the advantages of easy recovery of catalytic materials, high product purity, and high efficiency. At present, there is little research on photo-electric coenzyme regeneration based on microreactors, but the research on water decomposition based on microreactors is expected to promote its future progress. It is found that the oxidative decomposition of water can be realized by PEC [159,160], and holes can be trapped only by water as an electron sacrificial agent in this system [161,162], which effectively reduces the reverse transfer of electrons and helps to realize environmentally friendly, efficient, and economical photo-electric catalytic coenzyme regeneration without additional high-value amines as electron sacrificial agents [163]. Thakur et al. reported that they successfully prepared a paper-based photo-anode (PA) with good flexibility and stability by depositing polypyrrole (PPy) with electrical conductivity, high solar energy absorption, and a suitable cross-band (water oxidation–reduction) on laboratory filter paper, which produced a photocurrent density of about 9.5 mA/cm^2^ (1.23 V vs. RHE) in a three-electrode system (Figure 8E), and realized efficient and stable photo-electrochemical water decomposition [154] He et al. reported a metal–organic framework with an assembled bifunctional microreactor (Figure 8F). Under illumination, a photocurrent density of 3.50 Ma cm^–2^ can be obtained when it is integrated into a BiVO_4_ photo-anode, which proves that it can be applied to water decomposition to realize highly active solar-driven oxygen production [155]. It is also being studied to remove the recombination of photogenerated electrons and hole pairs in photo-electric catalytic reactions to improve catalytic efficiency. Wang et al. assembled ITO conductive glass, an adhesive layer, and BVO-modified ITO conductive glass to prepare a photo-electric catalytic microreactor (Figure 8G), and explored its influence on the catalytic performance of the microreactor by applying different bias potentials. The results showed that the catalytic performance of the reactor was better under negative bias conditions, and the problem of anoxia was successfully overcome through water decomposition [156]. In addition to the exploration and development of new efficient photocatalysts, the design and research of microreactors also promoted the improvement of photocatalytic performance. Kalamaras et al. designed a continuous photo-electrocatalytic microreactor with a planar sandwich structure (Figure 8H). When a double-layer α-Fe_2_O_3_/CuO is used as a photocathode, the highest photocurrent density of −1.0 mA cm^−2^ can be obtained under the irradiation of simulated sunlight, and formate and methanol products can be obtained at the same time, which shows the potential of converting common solar energy and CO_2_ into high-value chemical products by using water [157]. Different from the common planar immobilized microreactor, Suhadolnik et al. designed a continuous coil photo-electrocatalytic microreactor consisting of an anode fixed by photocatalyst and a cathode coil with applied voltage (Figure 8I). Compared with the conventional immobilized microreactor system, the photocatalytic material in this reactor is more firmly immobilized, with stronger photocatalytic activity, a simpler assembly and preparation steps, and a higher specific surface area and volume ratio. The test results show that this reactor displays excellent photo-electric catalytic water decomposition and the degradation of organic compounds such as caffeine [158].

## 5. Conclusions

This review mainly summarizes the research work on the photocatalysis, electrocatalysis, and photo-electrocatalysis of coenzyme regeneration in recent years, showing the environmental and economic advantages of photocatalysis and photo-electrocatalysis, and it further leads to the related research progress based on the microreactor and compares it with the conventional system. At present, the latter has started smoothly and obtained some research results. Compared with the conventional reaction system, the microreactor system integrating multiple technical units into a controllable micro-platform has many advantages, as follows: (1) It is small in size, and the inner diameter of its microchannel is less than 1 mm; (2) a plurality of technical units are highly controllable and integrated, so that a plurality of chemical reaction steps such as sample pretreatment, mixed reactions, product separation, and purification can be realized on one microfluidic chip, which simplifies the reaction steps and shortens the reaction time; (3) a high specific surface area and high mass and heat transfer efficiency greatly improve the reaction rate; and (4) the consumption of reagents is low, which reduces the cost of the chemical reaction and meets the requirements of industrialization. Using these advantages of the microreactor, the reaction rate of coenzyme regeneration can be significantly improved, the reaction time can be shortened, the reagent consumption can be reduced, the reaction cost can be reduced, and the industrial application of oxidoreductase can be promoted.

However, there are still some problems in the regeneration of coenzymes by photocatalysis, electrocatalysis, and photo-electrocatalysis based on microreactors, which need further exploration and solutions. For example, there are some problems in the regeneration of photocatalytic coenzymes, such as low catalytic efficiency, light-induced damage, light heating, and so on. There are some problems in the regeneration of electrocatalytic coenzymes, such as poor biocompatibility, high energy consumption, environmental pollution, and so on. There are also some problems in the regeneration of coenzymes by photo-electric catalysis, such as the following: the external circuit is not portable, the system is complicated, and the operation is complicated. The regeneration of various types of coenzymes in microreactors also has the problems of low flux and small scale. Although the problem can be solved to some extent through the parallel connection of multiple microreactors, other problems such as high cost and difficult heat dissipation arise. This situation shows that researchers still need to conduct in-depth research, optimize the size, stability, and sustainability of microreactors—while maintaining the above advantages of microreactors—and realize the large-scale application of microreactors.

In addition to the microreactor itself, the development and improvement of new catalysts, electrode modification methods, and new mediators are also key steps to improve the regeneration efficiency of coenzymes. For example, common photocatalysts such as g-C_3_N_4_ and TiO_2_ have some problems, such as a low specific surface area, poor stability, easy charge recombination, a low utilization rate of visible light, and most steps of electrode modification are cumbersome and costly. Conventional mediators need to improve their biocompatibility and selectivity. With the progress of material chemistry and biosynthesis research, researchers have developed methods or new materials such as catalyst surface functionalization, catalyst metal/nonmetal element doping, oxidized polymer modified electrodes, and new rhodium complexes to optimize these problems, improve catalytic performance, and obtain better catalytic efficiency.

In a word, researchers can further develop and explore the fields of photocatalysis, electrocatalysis, and photo-electrocatalysis coenzyme regeneration based on a new microreactor structure (expanding the specific surface area of the microreactor and increasing the fixed site of the catalyst, etc.), high-efficiency catalysts (catalyst functionalization, catalyst doping, etc.), electron transfer mediums (a new rhodium complex, etc.), electrodes (porous electrode, surface modified electrode, cocatalyst modified electrode, etc.), bias substitution means (series photovoltaic devices, etc.), reducing by-products (improving catalyst selectivity, etc.), coupling enzyme catalysis to generate high-value products (formic acid, etc.), and so on.

The new structural design and scale expansion of microreactors, the development of new efficient catalytic materials and mediators, and the optimization of electrode modification methods are effective ways and research focuses for the efficient development and utilization of solar energy to achieve convenient, fast, green and environmentally friendly, low-cost, and efficient microfluidic coenzyme regeneration. We believe that the research on coenzyme regeneration based on microreactors has great potential, and these problems will be overcome through the above-mentioned research and exploration, which will continuously improve the efficiency of coenzyme regeneration, help the industrial production of fine chemicals in which redox enzymes participate, and promote the development of the coenzyme regeneration microreactor and broaden its practical application fields.

## Figures and Tables

**Table 1:** Preparation method of microreactor.

Methods	Main Applications	Advantages	Disadvantage
Standard Lithography Technology	Silicon chip	1. Mature technology 2. High precision 3. Flexibility	1. The steps are cumbersome 2. Expensive equipment
Soft Lithography Technology	PDMS	1. Mature technology 2. Low cost 3. Flexibility	1. Long time 2. The preparation is complicated
3D Printing	High polymers	1. Simple operation 2. Flexible design 3. Environment-friendly 4. Complex microreactors can be prepared	1. Expensive equipment 2. The accuracy is limited 3. It takes a long time 4. The finished product has a low mechanical strength
Textile Technology	Cloth	1. Mature technology	1. Low accuracy 2. The types of applicable materials are limited
Laser-Induced Hydrophilization Technology	Cloth	1. High accuracy	1. The equipment requirements are higher. 2. The types of applicable materials are limited
Screen Printing Technology	Cloth	1. Simple operation 2. Mature technology 3. Convenient and quick 4. The equipment occupies a small area	1. Diffusivity of printed materials leads to low accuracy 2. Fewer applicable materials
Thermoforming Technology	Glass	1. The manufacturing process is simple 2. The preparation requirement is low 3. Time consumption is short	1. The finished product is not resistant to high temperature 2. Low accuracy
Spacer Technique	PDMS	1. A microreactor with a special internal structure can be prepared 2. No sealing operation is needed 3. No additional expensive equipment is needed	1. Low accuracy 2. The spacer causes loss to the base material 3. Spacer residue
Mechanical Processing	Glass	1. Simple operation 2. Low equipment requirements 3. Mature technology	1. It is not suitable for the processing of precise microstructures 2. Fewer applicable materials

**Table 2:** Difference between microreactor and conventional reactor system for coenzyme regeneration.

System	Conventional Reactor System	Microreactor System
Reagent consumption	Large reagent consumption (mL/L)	Low reagent consumption (μL)
Mass transfer and heat transfer efficiency	General mass transfer and heat transfer efficiency	High mass and heat transfer efficiency
Specific surface area	Low specific surface area	High specific surface area
Reaction efficiency	Low reaction efficiency	High reaction efficiency
Portability	Nonportability	Portability
Cost	Higher cost	Lower cost
Catalyst recovery	Not easy to recycle	Easy to recycle
